# An Update on Treatment of Genotype 1 Chronic Hepatitis C Virus Infection: 2011 Practice Guideline by the American Association for the Study of Liver Diseases

**DOI:** 10.1002/hep.24641

**Published:** 2011-10

**Authors:** Marc G Ghany, David R Nelson, Doris B Strader, David L Thomas, Leonard B Seeff

**Affiliations:** 1 Liver Diseases Branch, National Institute of Diabetes and Digestive and Kidney Diseases, National Institutes of HealthBethesda, MD; 2 Section of Hepatobiliary Disease, University of FloridaGainesville, FL; 3 Gastroenterology and Hepatology Division, Fletcher Allen Health Care, University of Vermont College of MedicineBurlington, VT; 4 Johns Hopkins Medical InstitutionBaltimore, MD; 5 The Hill GroupBethesda, MD

This practice guideline has been approved by the American Association for the Study of Liver Diseases (AASLD) and endorsed by the Infectious Diseases Society of America, the American College of Gastroenterology and the National Viral Hepatitis Roundtable.

## Preamble

These recommendations provide a data-supported approach to establishing guidelines. They are based on the following: (1) a formal review and analysis of the recently published world literature on the topic (MEDLINE search up to June 2011); (2) the American College of Physicians' *Manual for Assessing Health Practices and Designing Practice Guidelines*;^1^ (3) guideline policies, including the AASLD Policy on the Development and Use of Practice Guidelines and the American Gastroenterological Association's Policy Statement on the Use of Medical Practice Guidelines;^2^ and (4) the experience of the authors in regard to hepatitis C.

Intended for use by physicians, these recommendations suggest preferred approaches to the diagnostic, therapeutic, and preventive aspects of care. They are intended to be flexible, in contrast to standards of care, which are inflexible policies to be followed in every case. Specific recommendations are based on relevant published information. To more fully characterize the quality of evidence supporting recommendations, the Practice Guidelines Committee of the AASLD requires a Class (reflecting benefit versus risk) and Level (assessing strength or certainty) of Evidence to be assigned and reported with each recommendation (Table 1, adapted from the American College of Cardiology and the American Heart Association Practice Guidelines).^3^,^4^

**Table 1:** Grading System for Recommendations

Classification	Description
Class 1	Conditions for which there is evidence and/or general agreement that a given diagnostic evaluation procedure or treatment is beneficial, useful, and effective
Class 2	Conditions for which there is conflicting evidence and/or a divergence of opinion about the usefulness/efficacy of a diagnostic evaluation, procedure, or treatment
Class 2a	Weight of evidence/opinion is in favor of usefulness/efficacy
Class 2b	Usefulness/efficacy is less well established by evidence/opinion
Class 3	Conditions for which there is evidence and/or general agreement that a diagnostic evaluation, procedure/treatment is not useful/effective and in some cases may be harmful
Level of Evidence	Description
Level A	Data derived from multiple randomized clinical trials or meta-analyses
Level B	Data derived from a single randomized trial, or nonrandomized studies
Level C	Only consensus opinion of experts, case studies, or standard-of-care

## Introduction

The standard of care (SOC) therapy for patients with chronic hepatitis C virus (HCV) infection has been the use of both peginterferon (PegIFN) and ribavirin (RBV). These drugs are administered for either 48 weeks (HCV genotypes 1, 4, 5, and 6) or for 24 weeks (HCV genotypes 2 and 3), inducing sustained virologic response (SVR) rates of 40%-50% in those with genotype 1 and of 80% or more in those with genotypes 2 and 3 infections.^5-7^ Once achieved, an SVR is associated with long-term clearance of HCV infection, which is regarded as a virologic “cure,” as well as with improved morbidity and mortality.^8-10^ Two major advances have occurred since the last update of treatment guidelines for chronic hepatitis C (CHC) that have changed the optimal treatment regimen of genotype 1 chronic HCV infection: the development of direct-acting antiviral (DAA) agents^11-17^ and the identification of several single-nucleotide polymorphisms associated with spontaneous and treatment-induced clearance of HCV infection.^18^,^19^ Although PegIFN and RBV remain vital components of therapy, the emergence of DAAs has led to a substantial improvement in SVR rates and the option of abbreviated therapy in many patients with genotype 1 chronic HCV infection. A revision of the prior treatment guidelines is therefore necessary, but is based on data that are presently limited. Accordingly, there may be need to reconsider some of the recommendations as additional data become available. These guidelines review what treatment for genotype 1 chronic HCV infection is now regarded as optimal, but they do not address the issue of prioritization of patient selection for treatment or of treatment of special patient populations.

## Direct-Acting Antiviral Agents

There are multiple steps in the viral lifecycle that represent potential pharmacologic targets. A number of compounds encompassing at least five distinct drug classes are currently under development for the treatment of CHC. Presently, only inhibitors of the HCV nonstructural protein 3/4A (NS3/4A) serine protease have been approved by the Food and Drug Administration (FDA).

## Protease Inhibitors

The NS3/4A serine protease is required for RNA replication and virion assembly. Two inhibitors of the NS3/4A serine protease, boceprevir (BOC) and telaprevir (TVR), have demonstrated potent inhibition of HCV genotype 1 replication and markedly improved SVR rates in treatment-naïve and treatment-experienced patients.^12^,^13^,^16^,^17^ Limited phase 2 testing has shown that TVR also has activity against HCV genotype 2 infection but not against genotype 3.^20^ With regard to BOC, there are limited data indicating that it too, has activity against genotype 2 but also against genotype 3 HCV infection.^21^ However, at this time, neither drug should be used to treat patients with genotype 2 or 3 HCV infections, and when administered as monotherapy, each PI rapidly selects for resistance variants, leading to virological failure. Combining either PI with PegIFN and RBV limits selection of resistant variants and improves antiviral response.^15^

## Patients Who Have Never Received Therapy (Treatment-Naïve Patients)

### Boceprevir

**Fig. 1:**
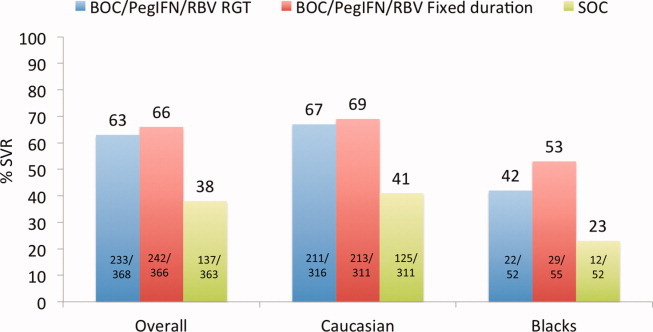
Sustained virological response (SVR) rates, overall and according to race, in treatment-naïve patients with genotype 1 chronic HCV infection: Boceprevir (BOC) plus peginterferon (PegIFN) and ribavirin (RBV) versus standard of care (SOC). All patients were first treated with PegIFN + RBV for 4 weeks as lead-in therapy followed by one of three regiments: (1) BOC/PegIFN/RBV RGT - triple therapy for 24 weeks provided HCV RNA levels were negative weeks 8 thorugh 24 – response guided therapy; those with a detectable HCV RNA level between weeks 8 and 24 received SOC for an additional 20 weeks; (2) BOC/PegIFN/RBV fixed duration - triple therapy for a fixed duration of 44 weeks; and (3) SOC - consisted of PegIFN and weight based RBV administered for 48 weeks.^12^

**Fig. 2:**
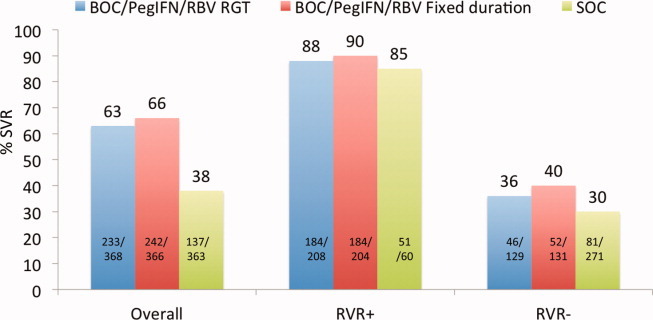
Sustained virological response (SVR) rates, overall and based on a rapid virological response (RVR, undetectable HCV RNA at week 8 [week 4 of triple therapy]) in treatment-naïve patients with genotype 1 chronic HCV infection: Boceprevir (BOC) plus peginterferon (PegIFN) versus standard of care (SOC). All patients were first treated with PegIFN + RBV for 4 weeks as lead-in therapy followed by one of three regiments: (1) BOC/PegIFN/RBV RGT - patients who achieved an RVR (undetable HCV RNA at week 8 [week 4 of triple therapy]) continued treatment for an additional 24 weeks (RGT - response guided therapy); if an RVR did not develop, treatment with triple therapy continued to week 28 followed by SOC treatment for 20 weeks. SOC treatment consisted of PegIFN and RBV administered for 48 weeks.^12^ Note that the combined numbers of RVR-positive and RVR-negative patients are not equivalent to the total number of patients enrolled, presumably because of missing HCV RNA values at the week 8 time point.

The *SPRINT-2* trial evaluated BOC in two cohorts of treatment-naïve patients: Caucasian and black patients.^12^ The number of patients in the black cohort was small in comparison to that of the Caucasian cohort and may have been insufficient to provide an adequate assessment of true response in this population. All patients were first treated with PegIFN alfa-2b and weight-based RBV as lead-in therapy for a period of 4 weeks, followed by one of three regimens: (1) BOC, PegIFN, and RBV that was administered for 24 weeks if, at study week 8 (week 4 of triple therapy), the HCV RNA level became undetectable (as defined in the package insert as <10-15 IU/mL), referred to as response-guided therapy (RGT); if, however, HCV RNA remained detectable at any visit from week 8 up to but not including week 24 (i.e., a slow virological response), BOC was discontinued and the patient received SOC treatment for an additional 20 weeks (2) BOC, PegIFN, and RBV administered for a fixed duration of 44 weeks; and (3) PegIFN alfa-2b and weight-based RBV alone continued for an additional 44 weeks, representing SOC therapy.^12^ The BOC dose was 800 mg, given by mouth three times per day with food. The overall SVR rates were higher in the BOC arms, (63% and 66% respectively) than in the SOC arm (38%), but differed according to race (Fig. 1). The SVR rates among Caucasian patients were 67% in the RGT, 69% in the fixed duration, and 41% in the SOC arms, respectively.^12^ In black patients, the SVR rates were 42% in the RGT, 53% in the fixed duration, and 23% in the SOC arms, respectively (Fig. 1).^12^ A total of 54% of Caucasian recipients of BOC experienced a rapid virological response (RVR; HCV RNA undetectable, <10-15 IU/mL at week 8, this interval selected because of the 4 week lead-in). By contrast, only 20% of black recipients of BOC experienced an RVR. Regardless of race, among those patients who became HCV RNA negative at week 8 (∼57% in both BOC arms and 17% in SOC arm), the SVR rates were 88% in the RGT arm, 90% in the fixed duration arm and 85% in the arm treated by SOC, compared to SVR rates of 36%, 40%, and 30%, respectively, if HCV RNA remained detectable at week 8 (Fig. 2).^12^

In subgroup analysis, SVR rates were higher in BOC-containing regimens across all the pretreatment variables that had been identified in previous studies to influence response to SOC therapy, including advanced fibrosis, race, and high pretreatment HCV viral load. Moreover, the SVR rate in subgroups was similar in both the RGT and fixed duration arms and therefore, the AASLD and the FDA support the use of RGT for treatment-naïve patients without cirrhosis. The FDA recommends that patients with compensated cirrhosis should not receive RGT, however, this is based on limited data and requires further study. Of note, if the virological response did not meet criteria for RGT, i.e., a slow virological response, the FDA recommends (based on modeling) triple therapy for 32 weeks preceded by the 4 weeks of SOC treatment), followed by 12 weeks of PegIFN and RBV alone; a strategy that differs from the phase 3 trial design. All therapy should be discontinued if the HCV RNA level is ≥100 IU/mL at week 12 or ≥10 to 15 IU/mL at week 24.

### Telaprevir

**Fig. 3:**
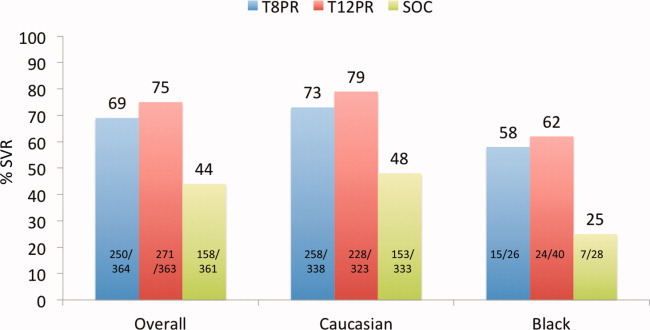
Sustained virological response (SVR) rates, overall and according to race, in treatment naïve patients with genotype 1 chronic HCV infection: Telaprevir (TVR) plus peginterferon and ribavirin (PR) treatment for 8 (T8PR) or 12 (T12PR) weeks versus standard of care (SOC). Patients in the triple therapy arms who developed an eRVR (extended rapid virological response; defined as undetectable HCV RNA at weeks 4 and 12) stopped treatment at week 24 (response-guided therapy, RGT); if eRVR did not develop, treatment continued to 48 weeks. SOC treatment consisted of PegIFN and RBV administered for 48 weeks.^16^

Two phase 3 trials evaluated the efficacy of TVR in combination with PegIFN alfa-2a and RBV in treatment-naïve patients with genotype 1 chronic HCV infection.^16^,^22^ Black patients were included but not as a separate cohort and were insufficient in number to provide an adequate assessment of true response in this population. In the *ADVANCE* trial, patients received TVR together with PegIFN and RBV for either 8 (T8PR) or 12 (T12PR) weeks followed by PegIFN and RBV alone in a response-guided paradigm.^16^ The TVR dose was 750 mg given by mouth every 8 hours with food (in particular, a fatty meal). Patients in the T8PR and T12PR groups who achieved an “extended RVR” (eRVR)—which for this drug was defined as undetectable (<10-15 IU/mL) HCV RNA levels at weeks 4 and 12—stopped therapy at week 24, whereas those in whom an eRVR did not occur received a total of 48 weeks of PegIFN and RBV. All patients in the control group received PegIFN and RBV therapy for 48 weeks. The overall SVR rates among patients in the T8PR and T12PR groups were 69% and 75%, respectively,^16^ compared with a rate of 44% in the control group (Table 2 and Fig. 3). Using the RGT approach, 58% and 57% of patients in the T12PR and T8PR groups, respectively, attained an eRVR, 89% and 83% of whom ultimately achieved an SVR.^16^ Thus, developing an eRVR appears to be the strongest predictor that an SVR will occur.

**Table 2:** Comparison of Protease Inhibitors in Combination with Peginterferon Alfa (PegIFN) and Ribavirin (RBV) in Treatment-Naive Subjects

Variable	Boceprevir (BOC)^12^	Telaprevir (TVR)^16^
Study design	RCT	RCT
4-Week lead-in PegIFN/RBV	Yes	No
Duration of triple therapy	24 or 44 weeks in combination with PegIFN/RBV*	12 weeks followed by 12 or 36 weeks PegIFN/RBV†
Response-guided therapy (RGT)	Yes	Yes
Eligible for response-guided therapy (%)	44	58
SVR (%)	BOC44/PR: 66	T8PR: 69
	BOC/PR/RGT: 63	T12PR: 75
	SOC: 38	SOC: 44
End of treatment response (%)	BOC44/PR: 76	T8PR: 81
	BOC/PR/RGT: 71	T12PR: 87
	SOC: 53	SOC: 63
Relapse (%)	BOC44/PR: 9	T8PR: 9
	BOC/PR/RGT: 9	T12PR: 9
	SOC: 22	SOC: 28
Treatment emergent resistance (%)	16	12
Adverse event more frequent in triple therapy arm compared to SOC	Anemia, dysgeusia	Rash, anemia, pruritus, nausea, diarrhea
Adverse events leading to treatment discontinuation (%)	NA	12
Serious adverse events study drug vs SOC (%)	11 vs 9	9 vs 7

NA, not available; PR, peginterferon plus ribavirin; RCT, randomized, controlled trial; SOC, standard of care; SVR, sustained virological response.

* All patients were first treated with PegIFN alfa-2b and weight-based RBV as lead-in therapy for a period of 4 weeks, followed by one of three regimens: (1) BOC/PR/RGT: BOC, PegIFN, and RBV that was administered for 24 weeks if, at study week 8 (week 4 of triple therapy), the HCV RNA level became undetectable (as defined in the package insert as <10-15 IU/mL), referred to as response-guided therapy (RGT); if, however, HCV RNA remained detectable at any visit from week 8 up to but not including week 24 (i.e., a slow virological response), BOC was discontinued and the patient received SOC treatment for an additional 20 weeks; (2) BOC44/PR: BOC, PegIFN, and RBV administered for a fixed duration of 44 weeks; and (3) SOC: PegIFN alfa-2b and weight-based RBV alone continued for an additional 44 weeks.

† Telaprevir (TVR) plus peginterferon and ribavirin (PR) treatment for 8 (T8PR) or 12 (T12PR) weeks versus standard of care (SOC). Patients in the T8PR and T12PR groups who achieved an “extended RVR” (eRVR), which for this drug was defined as undetectable (<10-15 IU/mL) HCV RNA levels at weeks 4 and 12, stopped therapy at week 24, whereas those in whom an eRVR did not occur received a total of 48 weeks of PegIFN and RBV. All patients in the control group received PegIFN and RBV therapy for 48 weeks.

SVR rates were higher in TVR-containing regimens compared to SOC treatment among patients with disease characteristics found previously to be associated with a poorer response to SOC treatment. Although few black patients and other difficult-to-treat patient populations were included in the TVR phase 3 trials, an improved SVR rate was observed regardless of race, ethnicity, or level of hepatic fibrosis. With regard to race, treatment with a TVR-based regimen significantly improved SVR rates in black patients (T8PR, 58% and T12PR, 62%) compared to the SVR rates achieved in those treated with the SOC regimen (25%) (Fig. 3). Moreover, the SVR rate was >80% among black patients who achieved an eRVR on a TVR-based regimen. A total of 62% of patients in the T12PR group and 53% in the T8PR group with advanced fibrosis achieved an SVR, the rate improving to >80% among those with an eRVR. In the T12PR group, the impact of high versus low viral load (>800,000 or <800,000 IU/mL) on SVR rates was minimal; the SVR rate was 74% in patients with a high viral load and 78% in those with a low viral load.

**Fig. 4:**
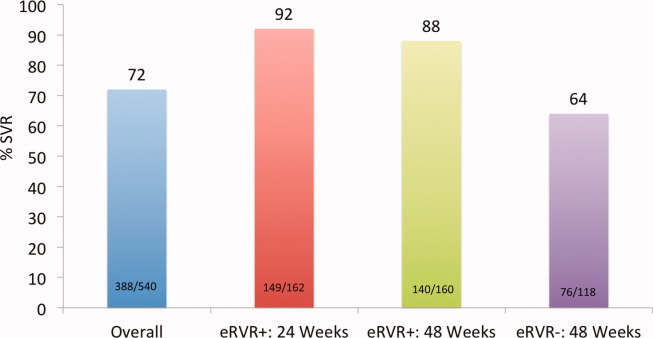
Sustained virological response (SVR) rates in treatment naïve patients with genotype 1 chronic HCV infection: Telaprevir (TVR) plus peginterferon and ribavirin (PR) results overall and among those who did or did not achieve an eRVR (extended rapid virological response; undetectable HCV RNA at weeks 4 and 12). Patients who achieved an eRVR were randomized at week 20 to receive an additional 4 or an additional 28 weeks of SOC therapy; those who did not develop an eRVR were not randomized and all received an additional 24 weeks of SOC therapy.^22^

The *ILLUMINATE* trial focused on defining the utility of RGT in patients with an eRVR. All patients received an initial 12 weeks of TVR-based triple therapy followed by PegIFN and RBV therapy alone.^22^ Those who achieved an eRVR were randomized at week 20 to receive either an additional 4 or an additional 28 weeks of PegIFN and RBV whereas those who failed to achieve an eRVR were not randomized and received an additional 28 weeks of PegIFN and RBV. The overall SVR rate for all patients was 72% (Fig. 4), similar to the 75% rate found in the *ADVANCE* trial.^22^ Among the 65% of patients who achieved an eRVR and received either an additional 4 or 28 weeks of PegIFN and RBV, SVR rates were 92% and 88%, respectively (Fig. 4). By contrast, the SVR rate was only 64% among patients who did not achieve an eRVR.^22^ These data suggest that a response-guided strategy based on eRVR permits a shortened duration of therapy without jeopardizing the SVR response rate and may be appropriate for up to two-thirds of patients with genotype 1 chronic HCV infection. The use of RGT may, however, be unsuitable for patients with cirrhosis, but at present the data are insufficient to guide management in this difficult-to-treat population. Therapy should be discontinued in all patients if HCV RNA levels are ≥1,000 IU/mL at weeks 4 or 12 and/or >10-15 IU/mL at week 24.

***Recommendations:***

1. ***The optimal therapy for genotype 1, chronic HCV infection is the use of boceprevir or telaprevir in combination with peginterferon alfa and ribavirin (Class 1, Level A).***
2. ***Boceprevir and telaprevir should not be used without peginterferon alfa and weight-based ribavirin (Class 1, Level A).***

***For Treatment-Naïve Patients:***

1. ***The recommended dose of boceprevir is 800 mg administered with food three times per day (every 7-9 hours) together with peginterferon alfa and weight-based ribavirin for 24-44 weeks preceded by 4 weeks of lead-in treatment with peginterferon alfa and ribavirin alone (Class 1, Level A).***
2. ***Patients without cirrhosis treated with boceprevir, peginterferon, and ribavirin, preceded by 4 weeks of lead-in peginterferon and ribavirin, whose HCV RNA level at weeks 8 and 24 is undetectable, may be considered for a shortened duration of treatment of 28 weeks in total (4 weeks lead-in with peginterferon and ribavirin followed by 24 weeks of triple therapy) (Class 2a, Level B).***
3. ***Treatment with all three drugs (boceprevir, peginterferon alfa, and ribavirin) should be stopped if the HCV RNA level is >100 IU/mL at treatment week 12 or detectable at treatment week 24 (Class 2a, Level B).***
4. ***The recommended dose of telaprevir is 750 mg administered with food (not low-fat) three times per day (every 7-9 hours) together with peginterferon alfa and weight-based ribavirin for 12 weeks followed by an additional 12-36 weeks of peginterferon alfa and ribavirin (Class 1, Level A).***
5. ***Patients without cirrhosis treated with telaprevir, peginterferon, and ribavirin, whose HCV RNA level at weeks 4 and 12 is undetectable should be considered for a shortened duration of therapy of 24 weeks (Class 2a, Level A).***
6. ***Patients with cirrhosis treated with either boceprevir or telaprevir in combination with peginterferon and ribavirin should receive therapy for a duration of 48 weeks (Class 2b, Level B).***
7. ***Treatment with all three drugs (telaprevir, peginterferon alfa, and ribavirin) should be stopped if the HCV RNA level is >1,000 IU/mL at treatment weeks 4 or 12 and/or detectable at treatment week 24 (Class 2a, Level B).***

## Patients Who Have Previously Received Therapy

**Fig. 5:**
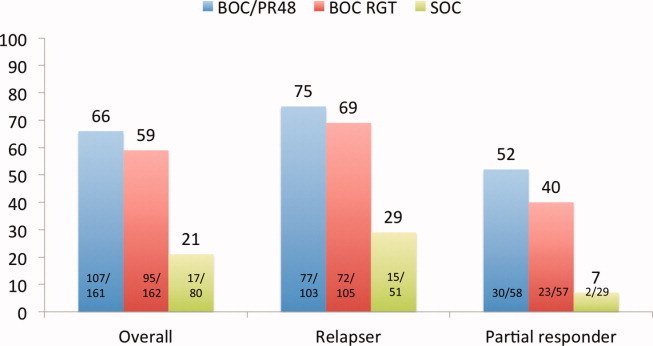
Sustained virological response (SVR) rates, overall and among relapsers and partial responders, in treatment experienced patients with genotype 1 chronic HCV infection: Boceprevir (BOC) plus peginterferon and ribavirin (PR) versus standard of care (SOC). All patients were first treated with PegIFN and RBV for 4 weeks as lead-in therapy followed by one of 3 regimens: (1) BOC/PR48 triple therapy for 44 weeks. (2) BOC RGT triple therapy for 32 weeks if an eRVR was achieved (undetecatble HCV RNA at week 8 and 12). If an eRVR was not achieved, but HCV RNA became undetectable at week 12, BOC was stopped at week 32 and patients received an additional 12 weeks of SOC treatment (total 48 weeks of therapy). (3) SOC treatment consisted of PegIFN and RBV administered for 48 weeks.^13^

**Fig. 6:**
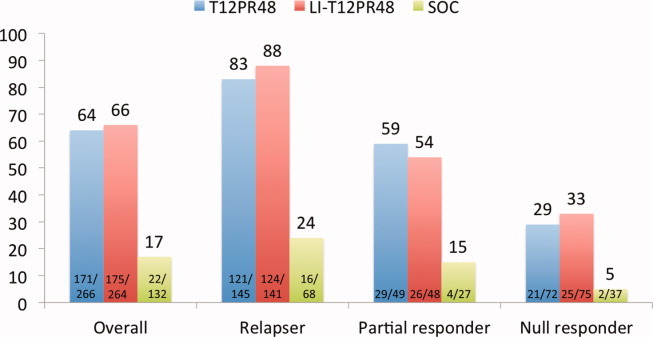
Sustained virological response (SVR) rates, overall and among relapsers, partial responders, and null responders, in treatment-experienced patients with genotype 1 chronic HCV infection. T12PR48: Telaprevir (TVR) plus peginterferon and ribavirin (PR) administered for 12 weeks followed by 36 PR for 12 weeks followed by PR for 32 weeks; SOC consisted of PegIFN and RBV administered for 48 weeks.^17^

Three categories have been defined for persons who had received previous therapy for CHC but who had failed the treatment. **Null responders** are persons whose HCV RNA level did not decline by at least 2 log IU/mL at treatment week 12; **partial responders** are persons whose HCV RNA level dropped by at least 2 log IU/mL at treatment week 12 but in whom HCV RNA was still detected at treatment week 24; and **relapsers** are persons whose HCV RNA became undetectable during treatment, but then reappeared after treatment ended. Taking these categories into account, phase 3 trials have been performed also in treatment-experienced patients with genotype 1 chronic HCV infection using BOC and TVR in combination with PegIFN and RBV. The BOC trial design included a 4-week lead-in phase of PegIFN and RBV and compared response-guided triple therapy (BOC plus PegIFN and RBV for 32 weeks; patients with a detectable HCV RNA level at week 8 received SOC for an additional 12 weeks) and a fixed duration of triple therapy given for 44 weeks (total 48 weeks of therapy), to SOC therapy.^13^ The TVR trial design consisted of three arms: in the first arm, patients received triple therapy for 12 weeks followed by SOC treatment for 36 weeks; in the second arm, patients received lead-in treatment with SOC for 4 weeks, followed by triple therapy for 12 weeks, ending with SOC treatment for 32 weeks; the third arm consisted of SOC treatment for 48 weeks.^17^ In both trials, an SVR occurred significantly more frequently in those who received the triple therapy regimens than in those who received the SOC therapy. In the BOC trial (*RESPOND-2 Trial*), the SVR rates were 66% and 59% in the two triple therapy arms compared to 21% in the control arm, prior relapsers achieving higher SVR rates (75% and 69%, respectively) than prior partial responders (52% and 40%, respectively) compared to the rates attained in the SOC arm (29% and 7%, respectively); null responders were excluded from this trial (Table 3 and Fig. 5).^13^ Similarly, the SVR rates in the TVR trial (*REALIZE Study*) were 64% and 66% in the TVR-containing arms (83% and 88% in relapsers, 59% and 54% in partial responders, and 29% and 33% in null responders) and 17% in the control arm (24% in relapsers, 15% in partial responders and 5% in null responders) (Fig. 6).^17^ Thus, the response to the triple therapy regimen in both the BOC and TVR trials was influenced by the outcome of the previous treatment with PegIFN and RBV which highlights the importance of reviewing old treatment records to document previous treatment response. In the BOC trial, the SVR rate was higher in those who were relapsers than in those who were partial responders. In the TVR trial also, the highest SVR rate occurred in prior relapsers, a lower rate in partial responders, and the lowest rate in null responders (defined as patients who had <2 log_10_ decline in HCV RNA at week 12 of prior treatment) (Table 3 and Fig. 6).^17^

**Table 3:** Comparison of Protease Inhibitors in Combination with Peginterferon Alfa (PegIFN) and Ribavirin (RBV) in Treatment-Experienced Patients

Variable	Boceprevir (BOC)^13^	Telaprevir (TVR)^17^
Study design	RCT	RCT
4-Week lead-in PegIFN/RBV	Yes	Yes/No*
Duration of triple therapy	32 or 44 weeks in combination with PegIFN and RBV^**^	12 weeks followed by 36 weeks of PegIFN and RBV^***^
Response-guided therapy (RGT)	Yes	No
Eligible for RGT (%)	46	NA
Prior response to PegIFN/RBV (%)		
Relapser	64	53
Partial responder	36	19
Null responder	NA	28
Efficacy, SVR (%)		
Relapser	BOC/PR48: 75	T12/PR48: 83
	BOC/RGT: 69	LI-T12/PR48: 88
	PR48: 29	PR48: 24
Partial responder	BOC/PR48: 52	T12/PR48: 59
	BOC/RGT: 40	LI-T12/PR48: 54
	PR48: 7	PR48: 15
Null responder	NA	T12/PR48: 29
		LI-T12/PR48: 33
		PR48: 5
Overall relapse (%)	12-15	NA
Relapser	NA	T12/PR48: 7
		LI-T12/PR48: 7
		PR48: 65
Partial responder	NA	T12/PR48: 21
		LI-T12/PR48: 25
		PR48: 0
Null responder	NA	T12/PR48: 27
		LI-T12/PR48: 25
		PR48: 60
Adverse events		
Discontinuation (%)	8-12	NA
SAE (%)	10-14	11-15
Adverse event more frequent in triple therapy arm	Anemia, dysgeusia	Rash, anemia, pruritus, nausea, diarrhea

NA, not available; PR, peginterferon plus ribavirin; RCT, randomized, controlled trial; SAE, serious adverse event; SVR, sustained virological response.

* A lead-in arm was included in the telaprevir retreatment trial but the FDA approved regimen did not include a lead-in strategy.

† The BOC trial design included a 4-week lead-in phase of PegIFN and RBV and compared response-guided triple therapy and a fixed duration triple therapy given for 44 weeks to peginterferon and ribavirin therapy. BOC/RGT response-guided therapy patients who achieved an eRVR (undetectable HCV RNA at week 8 [week 4 of triple therapy]) received an additional 24 weeks (total 32 weeks of therapy). If an eRVR was not achieved but HCV RNA became undetectable at week 12, BOC was stopped at week 32, and patients received an additional 12 weeks of SOC treatment (total 48 weeks of therapy). BOC/PR48: 4-week lead-in with peginterferon and ribavirin followed by a fixed duration of triple therapy for 44 weeks; PR48: PegIFN and RBV administered for 48 weeks.

‡ Telaprevir (TVR) plus peginterferon and ribavirin (PR) administered with and without a 4 week SOC treatment lead in versus standard of care (SOC). T12PR48: TVR administered for 12 weeks followed by 36 weeks of peginterferon and ribavirin; LI-T12/PR48: peginterferon and ribavirin for 4 weeks followed by TVR plus peginterferon and ribavirin for 12 weeks, followed by peginterferon and ribavirin for 32 weeks; PR48: peginterferon and ribavirin administered for 48 weeks.

Thus, the decision to re-treat patients should depend on their prior response to PegIFN and RBV, as well as on the reasons for why they may have failed, such as inadequate drug dosing or side effect management. Relapsers and partial responder patients can expect relatively high SVR rates to re-treatment with a PI-containing triple regimen and should be considered candidates for re-treatment. The decision to re-treat a null responder should be individualized, particularly in patients with cirrhosis, because fewer than one-third of null responder patients in the TVR trial achieved an SVR; there are no comparable data for BOC because null responders were excluded from treatment. In addition, a majority of null responders developed antiviral resistance. The FDA label, however, indicates that BOC can be used in null responders but, given the lack of definitive information from phase 3 data, caution is advised in the use of BOC in null responders until further supportive evidence becomes available. Accordingly, any potential for benefit from treating nonresponders must be weighed against the risk of development of antiviral resistance and of serious side effects, and the high cost of therapy.

Response-guided therapy, based on achieving an eRVR, was evaluated for retreatment in the BOC trial. Shortening the duration of therapy from the standard 48 weeks to 36 weeks in patients who received triple therapy and who achieved an eRVR (which for this drug was defined as HCV RNA negative weeks 8 through 20) did not significantly lower the SVR rate (59% for RGT versus 66% for fixed duration treatment).^13^ In patients with cirrhosis, however, the SVR rate was statistically lower in those who received RGT therapy than in those who were treated for the full 48-week duration (35% versus 77%, respectively).^13^ The emergence of BOC resistant variants was more common among patients who responded poorly to interferon treatment (<1 log decline in HCV RNA level) during the lead-in phase and who were treated with RGT compared to those with >1 log decline in HCV RNA level and treated for 48 weeks (32% and 8%, respectively).^13^ There are no comparable data for RGT using TVR. Nonetheless, SVR rates are at least as high in relapsers as in treatment-naïve patients, and TVR exposure is 12 weeks with both RGT and 48-week treatment options. Accordingly, although there are no direct data to support the recommendation that relapsers could be treated with TVR using an RGT approach, the FDA does endorse such a recommendation, as is the case for BOC.

## Utility of Lead-In

There is uncertainty about the benefit of a lead-in phase. Theoretically, a PegIFN and RBV lead-in phase may serve to improve treatment efficacy by lowering HCV RNA levels which would allow for steady-state PegIFN and RBV levels at the time the PI is dosed, thereby reducing the risk of viral breakthrough or resistance. In addition, a lead-in strategy does allow for determination of interferon responsiveness and on-treatment assessment of SVR in patients receiving either BOC or TVR. Patients whose interferon response is suboptimal, defined as a reduction of the HCV RNA level of less than 1 log during the 4-week lead-in, have lower SVR rates than do patients with a good IFN response during lead-in treatment.^12^ Nevertheless, the addition of BOC to poor responders during lead-in still leads to significantly improved SVR rates (28% to 38% compared with 4% if a PI is not added) and thus a poor response during the lead-in phase should not be used to deny patients access to PI therapy.

A direct comparison of the lead-in and non-lead-in groups in the BOC phase 2 study, however, did not show a significant difference in SVR rates for either the 28 week regimen, 56% and 54%, or the 48 week regimen, 75% and 67%, treated with and without lead-in, respectively.^11^ Combining data across all treatment groups in the phase 2 trial demonstrated a trend for a higher rate of virological breakthrough in the BOC-treated patients without a lead-in, 9%, than in those who received lead-in treatment, 4%, (*P* = 0.06). However, because all the phase 3 data were based on the lead-in strategy, until there is evidence to the contrary, BOC should be used with a 4-week lead-in. A lead-in strategy was not evaluated in the phase 3 TVR treatment-naïve trial, and therefore no recommendation can be made for this drug.

***Recommendations:***

***For treatment-experienced patients:***

1. ***Re-treatment with boceprevir or telaprevir, together with peginterferon alfa and weight-based ribavirin, can be recommended for patients who had virological relapse or were partial responders after a prior course of treatment with standard interferon alfa or peginterferon alfa and/or ribavirin (Class 1, Level A).***
2. ***Re-treatment with telaprevir, together with peginterferon alfa and weight-based ribavirin, may be considered for prior null responders to a course of standard interferon alfa or peginterferon alfa and/or weight-based ribavirin (Class 2b, Level B.)***
3. ***Response-guided therapy of treatment-experienced patients using either a boceprevir- or telaprevir-based regimen can be considered for relapsers (Class 2a, Level B for boceprevir; Class 2b, Level C for telaprevir), may be considered for partial responders (Class 2b, Level B for boceprevir; Class 3, Level C for telaprevir), but cannot be recommended for null responders (Class 3, Level C).***
4. ***Patients re-treated with boceprevir plus peginterferon alfa and ribavirin who continue to have detectable HCV RNA > 100 IU at week 12 should be withdrawn from all therapy because of the high likelihood of developing antiviral resistance (Class 1, Level B).***
5. ***Patients re-treated with telaprevir plus peginterferon alfa and ribavirin who continue to have detectable HCV RNA > 1,000 IU at weeks 4 or 12 should be withdrawn from all therapy because of the high likelihood of developing antiviral resistance (Class 1, Level B).***

## Adverse Events

Adverse events occurred more frequently in patients treated with PIs than in those treated with PegIFN and RBV therapy alone. In the BOC trials, anemia and dysgeusia were the most common adverse events, whereas in the TVR trials, rash, anemia, pruritus, nausea, and diarrhea developed more commonly among those who received TVR than who received SOC therapy.^12^,^16^ In the phase 3 TVR trials, a rash of any severity was noted in 56% of patients who received a TVR-based regimen compared to 32% of those who received a PegIFN and RBV regimen.^16^ The rash was typically eczematous and maculopapular in character, consistent with a drug-induced eruption. In most patients, the rash was mild to moderate in severity but was severe (involving >50% of the body surface area) in 4% of cases. The development of rash necessitated discontinuation of TVR in 6% and of the entire regimen in 1% of the cases. The Stevens Johnson Syndrome or Drug-Related Eruption with Systemic Symptoms (DRESS) occurred in <1% of subjects but at a higher frequency than generally observed for other drugs. The response of the rash to local or systemic treatment with corticosteroids and oral antihistamines is uncertain. Pruritus, commonly but not always associated with rash, was noted in ∼50% of patients who received TVR therapy.^16^

Anemia developed among recipients of both PIs. Hemoglobin decreases below 10 g/dL (grade 2 toxicity) occurred in 49% of patients who received a BOC regimen compared to 29% of those who received the SOC regimen, whereas 9% had a hemoglobin decline of <8.5 g/dL (grade 3 toxicity).^12^ Among patients treated with T12PR, hemoglobin levels of <10 g/dL were observed in 36% of patients compared to in 14% of patients who received SOC, and 9% had hemoglobin decreases to <8.5 g/dL.^16^ Because hematopoietic growth factors were not permitted during the TVR trials, there was a 5%-6% higher rate of treatment discontinuation among those who developed anemia than among those who did not. However, neither anemia nor RBV dose reduction adversely affected the SVR rate. Of note is that in the BOC trial, SVR rates in patients managed by RBV dose reduction alone were comparable to those in patients managed with erythropoietin therapy.^23^ Similarly, in the TVR trials, dose reduction of RBV had no effect on SVR rates, and therefore dose reduction should be the initial response to management of anemia.^24^ Because the duration of BOC therapy (24 to 44 weeks) is longer than the duration of TVR therapy (12 weeks), the frequency of anemia is likely to be greater in BOC-containing regimens, leading to more RBV dose reductions and consideration of erythropoietin use. However, the potential benefits of erythropoietin must be weighed against its potential side effects, the fact that its use in HCV therapy is not approved by the FDA, and its considerable cost. If a PI treatment–limiting adverse event occurs, PegIFN and RBV can be continued provided that an on-treatment response had occurred. There are no data to help guide substitution of one for the other HCV PI. If a patient has a serious adverse reaction related to PegIFN and/or RBV, the PegIFN and/or RBV dose should be reduced or discontinued. If either PegIFN and/or RBV are discontinued, the HCV PI should be stopped. Additional information on management of other adverse events can be found in the package insert.

## Drug–Drug Interactions

Because patients with CHC frequently receive medications in addition to those used to treat HCV infection, and because the PIs can inhibit hepatic drug-metabolizing enzymes such as cytochrome P450 2C (CYP2C), CYP3A4, or CYP1A, both BOC and TVR were studied for potential interactions with a number of drugs likely to be coadministered. These included statins, immune suppressants, drugs used to treat HIV coinfection, opportunistic infections, mood disorders, and drug addiction support medications. Both BOC and TVR, were noted to cause interactions with several of the drugs examined, either increasing or decreasing pharmacokinetic parameters. **It is particularly important, therefore, that the medical provider review this information as listed in the package insert for each of the drugs before starting treatment for CHC**. This information can be obtained at the FDA Web site: http://www.accessdata.fda.gov/scripts/cder/drugsatfda/index.cfm. Other helpful sites are: http//:222.drug-interactions.com and http://www.hep-druginteractions.org

## Viral Resistance and Monitoring

Emergence of antiviral-resistant variants during PI-based therapy has been observed during all trials and is associated with virological failure and relapse (Tables 2 and 3). Mutations that confer either high or low level resistance to BOC and TVR cluster around the catalytic site of the NS3/4A serine protease. Similar variants were detected in both BOC and TVR-treated subjects, suggesting that some degree of cross-resistance exists between the two PIs. In both phase 3 studies, sequence analysis of the NS3/4A region was performed in all subjects at baseline and for all subjects who failed to achieve an SVR. Antiviral resistant variants were detected in a small proportion of patients at baseline, 7% in the BOC studies and 5% in the TVR trials, but did not appear to impact response to either PI.^25^,^26^ Therefore, there is currently no clinical indication for baseline resistance testing.

Among treatment-naïve patients receiving a BOC regimen, antiviral resistant variants developing during treatment were observed overall in 16% of patients (Table 2).^12^ During treatment, TVR-associated antiviral variants occurred in 12% of treatment-naïve patients and 22% of treatment-experienced patients (Tables 2 and 3).^16^,^17^ A majority (80%-90%) of patients who experienced virological breakthrough or incomplete virological suppression on therapy, or virological relapse after discontinuation of PI therapy, were found to have antiviral resistant variants. In the BOC studies, poor response to interferon (<1 log decline in HCV RNA during the lead-in phase) was associated with a higher rate of development of resistance.^12^ Among TVR-treated patients, population sequencing has suggested that high-level resistance develops more commonly when virological failure occurs during the initial 12 weeks of treatment, whereas low-level resistance variants are more likely when virological failure occurs later, during treatment with PegIFN and RBV alone. These observations highlight the importance of response to interferon for the prevention of emergence of antiviral resistance.

The clinical significance of antiviral resistant variants that emerge during PI therapy is uncertain. In longitudinal follow-up of patients enrolled in phase 2 trials, BOC-resistant variants were detected in 43% of subjects after 2 years of follow-up. Similarly, among patients with documented TVR-resistant variants who were enrolled in the TVR phase 3 trials, 40% still had detectable resistant variants after a median follow-up period of 45 weeks.^27^ In general, the decline or loss of variants appears to be related to their level of fitness.

Further data are needed to determine whether selection of these variants during and after PI therapy affects subsequent treatment choices. In phase 3 studies, the emergence of resistant variants and virological breakthrough was more common in patients infected with HCV subtype 1a than 1b, a result of a higher genetic barrier required for selection of resistant variants in HCV subtype 1b compared to 1a.^28^ Thus, HCV subtyping may play a role in helping to select future treatment regimens and predict the development of resistance. Finally, minimizing development of compensatory mutations would involve early discontinuation of PI therapy when antiviral therapy is unlikely to succeed. Although viral stop rules varied widely in the phase 2 and 3 trials, week 4 and 12 time points on triple therapy are still key decision points for stopping therapy based on HCV RNA levels. Current data suggest that for patients receiving BOC, therapy should be stopped at week 12 if the viral level is >100 IU/mL or >10-15 IU/mL at treatment week 24 and, for TVR, therapy should be stopped at either week 4 or 12 if the viral level is >1,000 IU/mL or if week 24 HCV RNA is detectable.

***Recommendations:***

1. ***Patients who develop anemia on protease inhibitor-based therapy for chronic hepatitis C should be managed by reducing the ribavirin dose (Class 2a, Level A).***
2. ***Patients on protease inhibitor-based therapy should undergo close monitoring of HCV RNA levels and the protease inhibitors should be discontinued if virological breakthrough (>1 log increase in serum HCV RNA above nadir) is observed (Class 1, Level A).***
3. ***Patients who fail to have a virological response, who experience virological breakthrough, or who relapse on one protease inhibitor should not be re-treated with the other protease inhibitor (Class 2a, Level C).***

## Role of IL28B Testing in Decision to Treat and Selection of Therapeutic Regimen

The likelihood of achieving an SVR with PegIFN and RBV and of spontaneous resolution of HCV infection differ depending on the nucleotide sequence near the gene for IL28B or lambda interferon 3 on chromosome 19.^18^,^19^ One single-nucleotide polymorphism that is highly predictive is detection of the C or T allele at position rs12979860.^18^ The CC genotype is found more than twice as frequently in persons who have spontaneously cleared HCV infection than in those who had progressed to CHC. Among persons with genotype 1 chronic HCV infection who are treated with PegIFN and RBV, SVR is achieved in 69%, 33%, and 27% of Caucasians who have the CC, CT, and TT genotypes, respectively; among black patients, SVR rates were 48%, 15%, and 13% for CC, CT, and TT genotypes, respectively.^29^ The predictive value of *IL28B* genotype testing for SVR is superior to that of the pretreatment HCV RNA level, fibrosis stage, age, and sex, and is higher for HCV genotype 1 virus than for genotypes 2 and 3 viruses.^29^,^30^ There are other polymorphisms near the gene for IL28B that also predict SVR, including detection of the G or T allele at position rs8099917, where T is the favorable genotype, and essentially provides the same information in Caucasians as C at rs12979860.^31^,^32^

In one study, as well as in preliminary analyses of the phase 3 registration data, *IL28B* genotype remained predictive of SVR even in persons taking BOC or TVR.^33^ In Caucasian patients randomized in the *SPRINT 2* trial to take BOC for 48 weeks, SVR was achieved by 80%, 71%, and 59% of patients with CC, CT, and TT genotypes, respectively.^34^ In Caucasian patients randomized in the *ADVANCE* trial to take TVR for 12 weeks, SVR was achieved by 90%, 71%, and 73% of patients with CC, CT, and TT genotypes, respectively.^35^ *IL28B* genotype also predicts the likelihood of qualifying for RGT. In treatment-naïve Caucasian patients randomized in *SPRINT 2* to BOC, the week 8 HCV RNA threshold was achieved in 89% and 52% of patients with CC and CT/TT genotypes, respectively.^34^ In treatment-naïve Caucasian patients randomized in the *ADVANCE* study to TVR, eRVR was achieved in 78%, 57%, and 45% of patients with CC, CT, and TT genotypes, respectively.^35^ Although the IL28B genotype provides information regarding the probability of SVR and abbreviated therapy that may be important to provider and patient, there are insufficient data to support withholding PIs from persons with the favorable CC genotype because of the potential to abbreviate therapy and the trend for higher SVR rates observed in the TVR study. In addition, the negative predictive value of the T allele with PI-inclusive therapy is not sufficiently high to restrict therapy for all patients, because SVR was achieved by more than half of Caucasians with the TT genotype.^34^,^35^

In summary, these data indicate that *IL28B* genotype is a significant pretreatment predictor of response to therapy. Consideration should be given to ordering the test when it is likely to influence either the physician's or patient's decision to initiate therapy. There are insufficient data to determine whether *IL28B* testing can be used to recommend selection of SOC over a PI-based regimen with a favorable genotype (CC) and in deciding upon the duration of therapy with either regimen.

***Recommendation:***

1. ***IL28B genotype is a robust pretreatment predictor of SVR to peginterferon alfa and ribavirin as well as to protease inhibitor triple therapy in patients with genotype 1 chronic hepatitis C virus infection. Testing may be considered when the patient or provider wish additional information on the probability of treatment response or on the probable treatment duration needed (Class 2a, Level B).***

## Special Populations

There is a paucity of information for many of the subgroups with the greatest unmet need for treatment (e.g., patients coinfected with HIV and HCV, those with decompensated cirrhosis, and those after liver transplantation). Data from phase 1 and 2 trials have provided interim information that may guide related treatment issues. BOC and TVR undergo extensive hepatic metabolism, BOC primarily by way of the aldoketoreductase (AKR) system but also by the cytochrome P450 enzyme system, whereas TVR is metabolized only by the cytochrome P450 enzyme system, and the main route of elimination is via the feces with minimal urinary excretion. Thus, no dose adjustment of BOC or TVR is required in patients with renal insufficiency. No clinically significant differences in pharmacokinetic parameters were observed with varying degrees of chronic liver impairment in patients treated with BOC and therefore, no dosage adjustment of this drug is required in patients with cirrhosis and liver impairment. Although TVR may be used to treat patients with mild hepatic impairment (Child-Turcotte-Pugh class A, score 5 or 6), it should not be used in HCV-infected patients with moderate to severe hepatic impairment, because no pharmacokinetic or safety data are available regarding its use in such patients. As noted above, BOC and TVR are both inhibitors of CYP3A4, and concomitant administration of medications known to be CYP3A4 substrates should be done with caution and under close clinical monitoring. Pharmacokinetic interactions have particular implications in HIV-coinfected and transplant populations, where drug–drug interactions will complicate treatment paradigms, so that any use of BOC or TVR in transplant or HIV-coinfected populations of patients with HCV should be done with caution and under close clinical monitoring. TVR and BOC are not recommended for use in children and adolescents younger than 18 years of age, because the safety and efficacy has not been established in this population. Thus, whereas BOC and TVR have great promise for improved SVR in special populations, many complex treatment issues remain to be evaluated in further phase 2 and 3 testing.

## Abbreviations

AKR: aldoketoreductase
BOC: boceprevir
CYP: cytochrome P450
DAA: direct-acting antivirals
eRVR: extended rapid virological response
FDA: Food and Drug Administration
HCV: hepatitis C virus
IL28B: interleukin-28B
NS3/4A: HCV nonstructural protein 3/4A
PegIFN: peginterferon
PI: protease inhibitor
RBV: ribavirin
RGT: response-guided therapy
RVR: rapid virological response
SOC: standard of care
SVR: sustained virological response
TVR: telaprevir
